# Trends in all-cause pneumonia and otitis media in children aged <2 years following pneumococcal conjugate vaccine introduction in Colombia

**DOI:** 10.1080/21645515.2020.1805990

**Published:** 2020-09-23

**Authors:** Gabriel Carrasquilla, Alexandra Porras-Ramírez, Sandra Martinez, Rodrigo DeAntonio, Raghavendra Devadiga, Carla Talarico, Diana C. Caceres, Maria M. Castrejon, Patricia Juliao

**Affiliations:** a ASIESALUD, Bogota, Colombia; b Grupo de Medicina Comunitaria y Salud Colectiva, Universidad El Bosque, Bogotá, Colombia; c Centro de Vacunación Internacional, S A Cevaxin, Panama City, Panama; d GSK, Biometrics, Bangalore, India; e Vaccines, GSK, Rockville, USA; f Vaccines, GSK, Bogotá, Colombia; g Vaccines, GSK, Panama City, Panama

**Keywords:** *Streptococcus pneumoniae*, pneumonia, otitis media, PCV, PHiD-CV, incidence, mortality, Colombia

## Abstract

In Colombia, pneumococcal conjugate vaccines (PCVs) were implemented into the infant universal mass vaccination program in a stepwise manner; PCV-7 between 2009 and 2011 in different geographic regions/cities, with nationwide introduction of a 10-valent vaccine (PHiD-CV) in 2012. We aimed to describe trends in all-cause pneumonia mortality and overall mortality, and in the incidence of all-cause pneumonia and otitis media (OM) in Colombian children <2 y (y = years) of age, before and after PCV introduction. We obtained mortality and incidence data, nationally and for five major cities (Bogota, Medellin, Barranquilla, Cali and Cartagena) from 2005–2016 and 2008–2016, respectively, comparing mortality and incidence proportions in the post-PCV introduction period with those in the pre-PCV period. Overall mean reductions in all-cause pneumonia mortality was observed in the post-PCV period nationally (48.8%; 95%CI: 45.5–51.8%) and in four cities including Bogota (77.1%; 71.1–81.8%) and Medellin (56.4%; 44.1–65.9%); no substantial reduction was observed in Cartagena. Similar findings were observed for overall mortality. Reductions in all-cause pneumonia incidence were observed in Bogota (66.0%; 65.5–66.6%), Medellin (40.6%; 39.3–41.9%) and Cartagena (15.0%; 11.2–18.6%), while incidence increased in Barranquilla (78.5%; 68.4–89.2%) and Cali (125.5%; 119.2–132.0%). All-cause OM incidence fell in Medellin and Bogota (42.1–51.1%) but increased (95.8%) in Barranquilla. In conclusion, overall reductions in disease outcomes were observed following PCV introduction in most cities and nationwide. Decreasing trends in outcomes were observed prior to PCV introduction, and limited data points and data reporting issues may have influenced our results. (ClinicalTrials.gov: NCT02567747)

## Introduction

*Streptococcus pneumoniae* is a major cause of pneumococcal disease mortality and morbidity in children <5 y of age in Latin America and, more specifically, in those aged <2 y; invasive pneumococcal disease (IPD), pneumonia and otitis media (OM) are the chief causes of pneumococcal morbidity and mortality.^1–4^ Inclusion of pneumococcal conjugate vaccines (PCVs) in national childhood immunization programs (NIPs) is recommended by the World Health Organization (WHO)^5^ and their introduction in Latin America has substantially changed disease burden in the region.^6^ Initially, some countries introduced the seven-valent conjugate vaccine (PCV-7, *Prevnar®*, Pfizer, including serotypes 4, 6B, 9V, 14, 18C, 19F, and 23F) which was replaced in infant universal mass vaccination (UMVs) programs by higher-valent PCVs with broader serotype coverage, e.g., pneumococcal non-typeable *Haemophilus influenzae* protein D conjugate vaccine (PHiD-CV, *Synflorix™*, GSK, which includes serotypes 1, 4, 5, 6B, 7F, 9V, 14, 18C, 19F, and 23F) or a 13-valent PCV (PCV-13, *Prevnar®*13, Pfizer, which includes serotypes 1, 3, 4, 5, 6A, 6B, 7F, 9V, 14, 18C, 19A, 19F, and 23F).^6^

**Figure 1.**
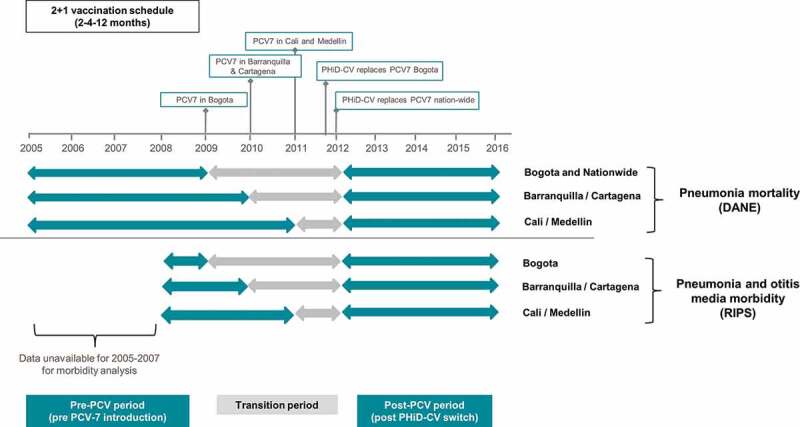
PCV introduction and study analysis time period

In Colombia, PCV-7 was introduced into the infant UMV program in 2009 with a 2 + 1 schedule at 2, 4, and 12 months of age. Implementation of PCV-7 was staggered across specific geographic areas and large metropolitan areas (including Bogota DC) in 2009, with further expansion to additional departments in 2010 (including the cities of Barranquilla and Cartagena) and then across the whole country in 2011 (including the cities of Cali and Medellin). At the end of 2011, PCV-7 was replaced by PHiD-CV in Bogota, followed by nationwide implementation in January 2012 (Figure 1); given as a 2 + 1 schedule at 2, 4, and 12 months of age.

•PCV7: 7-Valent Pneumococcal Conjugate Vaccine; UMV: Universal Mass Vaccination; PHiD-CV: Pneumococcal polysaccharide protein D-conjugate vaccine.

Evaluating changes in disease epidemiology following PCV introduction is recommended.^7,8^ While the benefit of PCV introduction in reducing IPD in Colombia has been reported,^9^ data on its impact on all-cause pneumonia and OM are currently limited. The aim of the present study is to report on the trends of all-cause pneumonia incidence and mortality and incidence of all-cause OM in Colombian children <2 y of age in five major metropolitan areas (Bogota DC, Medellin, Barranquilla, Cartagena, and Cali), before and after the introduction of PCVs in the NIP program. The impact on pneumonia mortality and overall all-cause mortality at a national level is also evaluated (Figure 1). We also estimated PCV vaccine coverage in these cities and nationwide.

## Methods

### Study design and data sources

We conducted a retrospective ecological study using secondary data sources to assess trends in all-cause pneumonia mortality and overall mortality, and in all-cause pneumonia and OM morbidity, before and after introduction of PCVs among children <2 y of age in Colombia. Mortality outcomes were evaluated nationally and across five cities within Colombia (Bogota, Barranquilla, Cartagena, Cali and Medellin), while all-cause pneumonia and OM incidence data were obtained from five cities (but not nationwide). These cities were selected as data reporting systems and were considered robust enough to allow adequate data capture for disease outcomes and for population and vaccination coverage estimates, along with less uncertainty in key factors such as PCV introduction date within the UMV.

Population size estimates and the number of deaths due to all-cause pneumonia (ICD-10: J12–J18) were obtained from the Colombian National Administrative Department of Statistics database (DANE) for 2005–2016.^10^ Annual number of deaths due to all-cause pneumonia as the underlying cause, and numbers of deaths due to any cause are presented in Supplementary Tables 2 and 3. Data on the number of cases due to all-cause pneumonia (ICD-10: J12–J18) and OM (ICD-10: H65–H66) were obtained from the Health Service Provider Individual Registration System (RIPS)^11^ for 2008–2016 (Supplementary Tables 4 and 5). Note that for both of these health epidemiology and reporting systems (DANE and RIPS) are subject to some element of under-reporting and also to variation in quality reporting in different administrative departments in Colombia. The DANE database records deaths on the basis of death certification and is considered a relatively robust source of data for mortality data, including all-cause pneumonia mortality.^12^ The RIPS database monitors health-care delivery and insurance claims, for both hospital and outpatient care, and is subject to greater uncertainty. Unlike DANE which reports on the overall population, previous studies (from 2012) have shown the RIPS database covers an estimated 50% of the population,^13^ and underreporting is well recognized,^13,14^ with perhaps only 35% of all pneumonia hospitalizations being reported.^12^ Relative differences in the reporting between hospital and outpatient care may also exist, with possibly substantial variation in data quality in different administrative departments, which may vary year-by-year.^13^

Data on vaccination coverage in infants <1 y who received a third dose of PCV were obtained from the National Immunization Program (PAI) database^15^ for each year for 2009–2016. All study data were obtained in an aggregated and anonymized format.

### Data analysis

Annual all-cause pneumonia mortality (per 100,000 children) and all-cause pneumonia and OM incidence (per 1000 children) estimates were calculated along with their 95% CIs (exact Poisson or asymptotic depending on estimate number) and plotted for the overall study periods; 2005–2016 for pneumonia mortality, and 2008–2016 for pneumonia and OM incidence (as data from 2005 to 2007 were not available from RIPS for the morbidity analyses). For mortality and incidence estimations, we made no adjustments to account for under-reporting of pneumonia or OM, the mortality and incidence rates we report are crude unadjusted rates.

For assessing the effects PCV on mortality and incidence we calculated mean annual estimates across three distinct vaccination periods: pre-PCV period, a transition period when there was some use of PCV7 and some use of PHiD-CV, and a post-PCV period when the switch to PHiD-CV was fully implemented across the country. For all outcomes and cities, the period following UMV introduction with PHiD-CV at a national level (2012–2016) was used as the post-PCV period. Due to the staggered implementation of PCV-7 and PHiD-CV introduction, which varied in the five different cities, and limitations in data availability from different data sources, the pre-PCV introduction period and the transition period also varied, as illustrated in Figure 1.

From these data, we calculated the percent reduction in all-cause pneumonia mean annual mortality (and all-cause mortality) proportions and all-cause pneumonia and OM mean annual incidence proportions in the post-PCV period compared to the pre-PCV period proportions, using the formula (% reduction = (mortality or incidence proportion [pre-vaccination period] – mortality or incidence proportion [post-vaccination period])/mortality or incidence proportion [pre-vaccination period] *100); where the pre-vaccination period was the pre-PCV-7 introduction period; and the post-vaccination period was the period following PHiD-CV introduction at a national level. Negative binomial regression was used to assess overall trends within the study period for both morbidity and mortality data. The model was calculated by log (No. of cases [or death]) = log (population projections) + intercept + β1 Year. The model output included the percent reduction in pneumonia annual mortality proportion for the overall study periods along with 95% CI and corresponding *p*-values.

Vaccine coverage for the third dose of PCVs (which reflects receipt of the third dose of the recommended 2 + 1 schedule for PCVs) was calculated using the number of third doses administered as reported in the PAI database divided by the population size estimate for children 1 y of age provided by the DANE database for each city. All analyses were carried out using SAS v.9.3 software.^16^

## Results

### Study population

In 2016, the estimated annual population of children <2 y in these five cities was 442,474: Bogota (243,156), Barranquilla (38,177), Cartagena (32,312), Cali (70,807), and Medellin (58,022), which corresponds to approximately 25% of the overall <2 y old national population in 2016 (Supplementary Table 1).

### All-cause pneumonia mortality and overall mortality

Between 2005 and 2016, 5,416 deaths were reported in children aged <2 y in Colombia due to all-cause pneumonia (ICD-10: J12–J18), with a total of 1,783 (32.9%) deaths reported across the 5 selected cities. In 2016 a total of 337 all-cause pneumonia deaths were reported nationwide, and 79 were reported across all 5 cities (Supplementary Table 2). A total of 115,726 deaths due to any cause were reported in children <2 y nationwide in the same period (2005–2016), with 19,185 reported across all 5 cities. In 2016, 7,296 deaths were reported nationally and 2,891 in the 5 cities (Supplementary Table 3).

**Figure 2.**
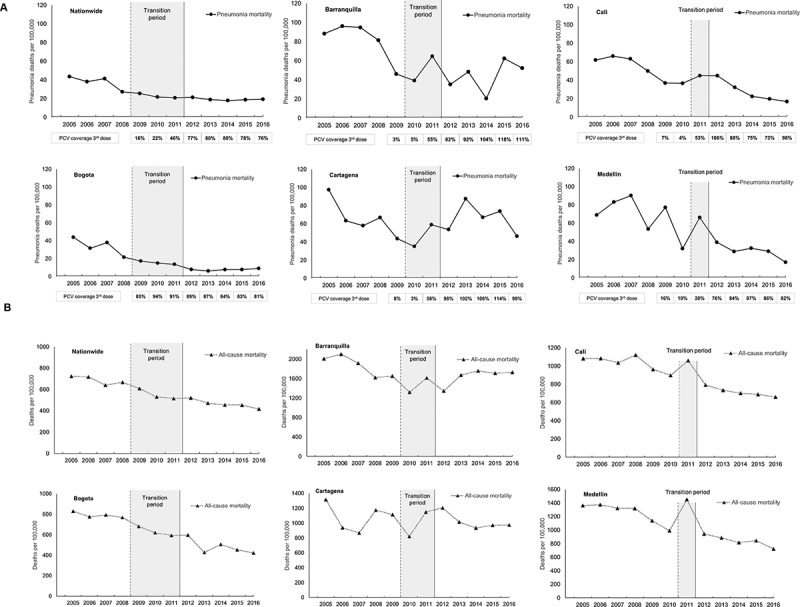
Annual mortality due to all-cause pneumonia (a) and annual all-cause mortality (b) in children <2 y of age (2005–2016)

All-cause pneumonia mortality and overall mortality rate estimates in children aged <2 y varied across cities with the highest burden of these deaths observed in Barranquilla and Cartagena and the lowest burden observed in Bogota (Table 1). Declining trends in all-cause pneumonia mortality were observed in Bogota, Barranquilla, Cali, Medellin and at a national level since 2005 (Figure 2 and Supplementary Table 6). A consistent pattern of decline of all-cause pneumonia mortality was observed at a national level and in Bogota (Table 1 and Figure 2). Declining trends were also observed in Cali, where a surge was observed in the PCV transition period (in 2011) with a subsequent consistent decline from 2012–2016, and for Medellin where mortality fluctuated prior to post-PCV period and a decline observed thereafter. In Barranquilla, an overall decline was also observed, though estimates fluctuated substantially in the post-PCV introduction period. For Cartagena, rates fluctuated throughout the study period (Table 1 and Figure 2). Trends in all-cause mortality (nationally and by city) were broadly similar to that seen for all-cause pneumonia mortality (Supplementary Table 6).

**Table 1.** **%** reductions in all-cause pneumonia mortality and overall all-cause mortality in post-PCV period compared to pre-PCV introduction period in children <2 y by vaccine period in selected cities and nationwide

	All-cause mortality and pneumonia mortality (deaths per 100,000 persons)								
	Overall period		Pre-PCV period (pre PCV-7 introduction)		Transition period		Post-PCV period (post PHiD-CV switch)		
	Years	Mean period estimate	Years	Mean period estimate	Years	Mean period estimate	Years	Mean period estimate	% Reduction (95%CI)*
**Pneumonia mortality**									
National	2005–2016	26.2	2005–2008	37.6	2009–2011	22.7	2012–2016	19.3	48.8 (45.5, 51.9)
Bogota DC	2005–2016	18.2	2005–2008	33.8	2009–2011	15.4	2012–2016	7.8	77.1 (71.1, 81.8)
Barranquilla	2005–2016	61.5	2005–2009	81.6	2010–2011	52.0	2012–2016	43.7	46.4 (30.5, 58.7)
Cartagena	2005–2016	62.8	2005–2009	66.0	2010–2011	47.1	2012–2016	65.8	0.3 (– 29.7, 23.4)
Cali	2005–2016	41.3	2005–2010	52.4	2011	45.0	2012–2016	27.3	47.8 (33.8, 58.9)
Medellin	2005–2016	51.5	2005–2010	67.5	2011	66.3	2012–2016	29.5	56.4 (44.1, 65.9)
**All-cause mortality**									
National	2005–2016	560.1	2005–2008	688.1	2009–2011	552.0	2012–2016	464.1	32.6 (31.7, 33.5)
Bogota DC	2005–2016	620.6	2005–2008	792.0	2009–2011	631.9	2012–2016	481.2	39.2 (37.1, 41.3)
Barranquilla	2005–2016	1707.9	2005–2009	1862.7	2010–2011	1466.0	2012–2016	1640.8	11.9 (7.7, 15.9)
Cartagena	2005–2016	1041.3	2005–2009	1082.7	2010–2011	939.9	2012–2016	1021.4	5.7 (–0.8, 11.7)
Cali	2005–2016	901.3	2005–2010	1030.7	2011	1060.1	2012–2016	716.4	30.5 (27.0, 33.8)
Medellin	2005–2016	1097.9	2005–2010	1251.4	2011	1452.7	2012–2016	843.5	32.6 (29.2, 35.8)

* % reduction = (mortality proportion [pre-vaccination period] – mortality proportion [post-vaccination period])/mortality proportion [pre-vaccination period] *100); Wald-type 95% confidence intervals (CIs) were calculated using Poisson regression.

•No legend.

Percentage reductions in all-cause pneumonia mortality in the post-PCV introduction period compared with the period prior to any PCV introduction are shown in Table 1 (and Supplementary Figure 1). Reductions were observed at a national level (48.8%; 95%CI: 45.5–51.8%), and in 4 of the 5 cities (Bogota, Medellin, Cali, and Barranquilla). The greatest reductions were observed in Bogota with all-cause pneumonia mortality falling by 77.1% (95%CI: 71.1–81.8%), followed by Medellin (56.4%; 95%CI: 44.1–65.9%), Cali (47.8%; 95%CI: 33.8–58.9%) and Barranquilla (46.4%; 95%CI: 30.5–58.7%); no statistically significant reductions in all-cause pneumonia mortality was observed in Cartagena. Reductions in all-cause mortality were also observed, nationwide (32.6%; 95%CI: 31.7–33.5%) and in most cities, including Bogota (39.2%; 95%CI: 37.1–41.3%) and Medellin (32.6%; 95%CI: 29.2–35.8%); no statistically significant reduction in all-cause mortality was found for Cartagena (Table 1).

### All-cause pneumonia incidence

**Figure 3.**
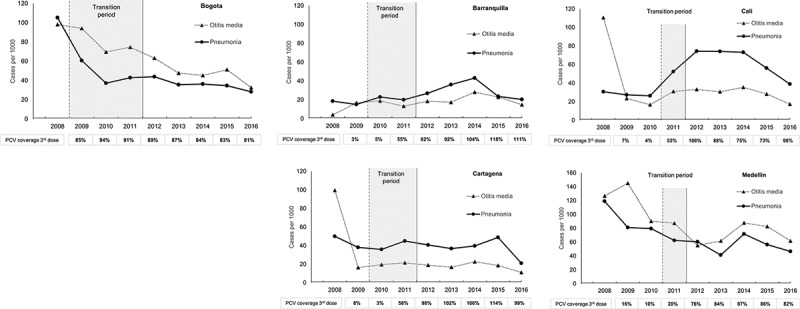
Annual incidence of pneumonia and otitis media in children <2 y of age (2008–2016)

Between 2008 and 2016, a total of 190,881 cases of all-cause pneumonia were reported in children <2 y in the 5 selected cities, with 53.2% (101,661 cases) reported for Bogota (Supplementary Table 4). Substantial variation in the incidence of all-cause pneumonia cases was observed during the 2008–2016 period in the different cities (Figure 3 and Table 2).

**Table 2.** Percentage reductions in all-cause pneumonia incidence and otitis media incidence in post-PCV period compared to pre-PCV introduction period in children <2 y by vaccine period in selected cities

	All-cause pneumonia Incidence and otitis media incidence (cases per 1000 persons)								
	Overall period		Pre-PCV period (pre PCV-7 introduction)		Transition period		Post-PCV period (post PHiD-CV switch)		
	Years	Mean period estimate	Years	Mean period estimate	Years	Mean period estimate	Years	Mean period estimate	% Reduction (95%CI)*
**Pneumonia Incidence**									
Bogota DC	2008–2016	47.0	2008	105.3	2009–2011	46.9	2012–2016	35.7	66.0 (65.5, 66.6)
Barranquilla	2008–2016	25.1	2008–2009	16.9	2010–2011	21.6	2012–2016	30.1	– 78.5 (–89.2, – 68.4)
Cartagena	2008–2016	39.6	2008–2009	44.0	2010–2011	40.5	2012–2016	37.4	15.0 (11.2,18.6)
Cali	2008–2016	50.5	2008–2010	28.1	2011	52.5	2012–2016	63.5	–125.5 (–132.0, – 119.2)
Medellin	2008–2016	68.7	2008–2010	93.1	2011	62.5	2012–2016	55.4	40.6 (39.3, 41.9)
**Otitis Media Incidence**									
Bogota DC	2008–2016	63.9	2008	98.2	2009–2011	79.4	2012–2016	48.0	51.1 (50.3, 51.8)
Barranquilla	2008–2016	17.1	2008–2009	10.4	2010–2011	16.1	2012–2016	20.4	–95.8 (–110.8, – 81.9)
Cartagena	2008–2016	27.5	2008–2009	58.0	2010–2011	20.5	2012–2016	17.7	ND
Cali	2008–2016	36.3	2008–2010	50.1	2011	31.1	2012–2016	29.1	ND
Medellin	2008–2016	88.7	2008–2010	120.6	2011	87.3	2012–2016	69.8	42.1 (41.0, 43.2)

* % reduction = (incidence proportion [pre-vaccination period] – incidence proportion [post-vaccination period])/incidence proportion [pre-vaccination period] *100); Wald-type 95% confidence intervals (CIs) were calculated using Poisson regression. ND, not done due to large biases observed with outliers and possible underreporting in the dataset.

•No legend.

Declining trends in the incidence of all-cause pneumonia were observed in Bogota, Medellin and Cartagena since 2008 (Figure 3 and Supplementary Table 6). In Bogota, a marked decline was observed in the pre-PCV and transition periods, followed by a lower decline in the post-PCV period. In Medellin, a decline was observed in the pre-PCV and transition periods, and the first 2 y of the post-PCV period, where the incidence proportion of all-cause pneumonia increased in 2014 and declined shortly after. In contrast, for Cali, the overall trend was that of increasing all-cause pneumonia incidence, although a decline was observed in 2015 and 2016 (Figure 3 and Supplemental Table 6). No defined overall trends were observed in Cartagena and Barranquilla.

Comparisons of the incidence proportions of all-cause pneumonia in the periods pre- and post-PCV introduction are shown in Table 2 (and Supplementary Figure 1). Substantial reductions were observed in Bogota (66.0%; 95% CI; 65.5–66.6%) and Medellin (40.6%; 95%CI; 39.3–41.9%) with a lower but significant reduction observed in Cartagena (15.0%; 95% CI; 11.2–18.6%). In contrast, an increase in all-cause pneumonia incidence in the post-PCV period compared with the pre-PCV period was observed in Barranquilla and Cali with increases of 78.5% (95%CI: 68.4–89.2%) and 125.5% (95%CI: 119.2–132.0%) respectively.

### All-cause otitis media incidence

A total of 217,356 all-cause OM cases in children <2 y were reported in the RIPS database between 2008 and 2016 (Supplementary Table 5). Bogota, the largest city in this study (Supplementary Table 1), had the greatest number of OM cases reported. In Cali and in Cartagena, more cases were seen in 2008 than in later years, while in contrast, fewer cases were reported in Barranquilla in 2008 than in subsequent years (Supplementary Table 5).

Variations in incidence for OM were observed between the five cities with the highest incidence reported in Medellin and the lowest incidence reported in Barranquilla and Cartagena during the analysis period (Figure 3 and Table 2). An overall declining trend in OM incidence was observed in four of the five cities, the exception being Barranquilla (Figure 3 and Supplemental Table 4). A consistent declining incidence was observed in Bogota and Medellin. For Cartagena and Cali, incidence dropped after 2008, with subsequent declining incidence observed in both cities. For Barranquilla, no overall trend was observed across the study period (Figure 3 and Supplementary Table 4).

Comparisons of the mean OM incidence in the pre- and post-PCV periods are shown in Table 2 (and Supplementary Figure 1). Reductions in the mean all-cause OM incidence were observed in Bogota (51.1%; 95%CI: 50.3–51.8%) and Medellin (42.1%; 95%CI: 41.0–43.2%). In contrast, an increase in the mean OM incidence (95.8%; 95%CI: 81.9–110.8%) was observed in Barranquilla. For Cartagena and Cali, observed outliers in the first year of data analysis (2008) as well as possible data reporting issues in the following years may have resulted in biases; as such, percent reduction analyses in the mean all-cause OM incidence were not conducted for these two cities.

### Vaccine coverage

**Figure 4.**
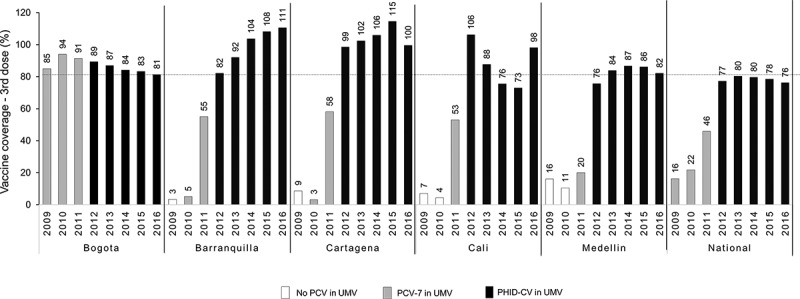
PCV vaccination coverage across the study period (2009–2016)

**Figure 5.**
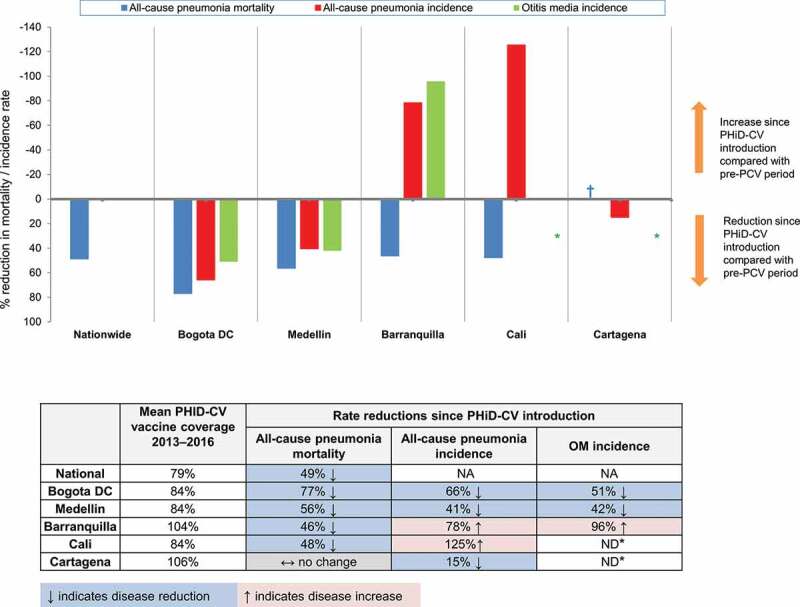
Reductions in all-cause pneumonia and otitis media

Figure 4 shows coverage with the third dose of the recommended 2 + 1 PCV schedule (given to infants at 12 months of age) in the five cities as well as at the national level. Vaccine coverage rates varied across cities during the earlier part of the vaccine introduction period (between 2009 and 2011). Of the 5 cities, only Bogota DC achieved and maintained >80% vaccination coverage since initial introduction, with 94% uptake in 2010, although reported coverage has since declined each year (to 81.3% in 2016). Uptake of PCV-7 in other cities was low between 2009 and 2011 but following PHiD-CV introduction, coverage has increased. The mean vaccine coverage across the 2012–2016 period at a national level was 79%, driven in part by lower uptake out with the 5 major cities (data not shown). In the five cities, mean vaccine coverage for this period ranges from 84% (in Bogota, Medellin and Cali) to over 100% in Barranquilla and Cartagena (Figure 5). In Cali, while uptake in 2012 was 100%, this subsequently fell (to 73% in 2015) and then increased to 98.1% in 2016. For those years where coverage estimates are above 100% in some cities, which may indicate that other factors, e.g.,, underestimation of population size due to population migration may have had some influence.

•No legend.

•Data represents percent reductions in mortality/incidence in the period following PHiD-CV introduction compared with those prior to use of any PCV. Below is a schematic representation of the pattern of disease reduction since PHiD-CV introduction and the mean vaccine coverage across 2013–2016. •NA, not available; nationwide pneumonia and otitis media (OM) incidence data were not available•* ND, not done; due to substantial biases observed with outliers and possible underreporting of otitis media, these cities were not included in the formal analyses for incidence reductions. † For Cartagena, reductions in pneumonia mortality were minimal.

## Discussion

We evaluated changes in reducing all-cause pneumonia mortality and the incidence of all-cause pneumonia and OM in Colombian children <2 y of age across 2005–2016. We compared disease estimates in all-cause pneumonia incidence and mortality and all-cause OM incidence in the period following PCV introduction and the period prior to any PCV-introduction, and evaluated trends across the study period in five major cities. Overall, we found heterogeneous patterns of potential PCV impact across disease outcomes.

As we discuss later below, our results show a variable pattern of all-cause pneumonia and OM epidemiology across the five cities (Figure 5), which in part may reflect inherent limitations in use of the case notifications reported in the RIPS claims database for epidemiological surveillance. Before we do so it is worth considering certain aspects of our pneumonia mortality and pneumonia and OM morbidity data in turn.

On average, reductions in all-cause pneumonia mortality were found post-PCV introduction at a national level (49%) and in Bogota (77%), Medellin (56%), Cali (48%), and Barranquilla (46%). Reductions in all-cause mortality were also observed, nationwide and in these four cities, although these were of a lower magnitude (ranging from 11.9% to 39.6%). It must be noted that reductions in all-cause pneumonia mortality began prior to PCV UMV introduction, and year-to-year variation in all-cause pneumonia mortality was observed (Figure 2a). In one such example, in Cartagena, the observed trend in all-cause pneumonia mortality, with a decline between 2005 − 2010, followed by an increase (2010–2013) and subsequent decline (with a broadly similar trend in all-cause mortality) may in part be explained by certain events such as severe flooding in the area in 2010/11, in which substantial population displacement and pressure on the authorities to settle displaced persons in areas served by weakened local healthcare infrastructure have been reported.^17^ It is possible that this may have contributed to greater mortality seen in the earlier part of the period following PHiD-CV introduction. It must also be recognized that temporal variations in other etiologies contributing to all-cause pneumonia mortality may influence the temporal mortality trends we observed.

Our observations for pneumonia mortality are somewhat consistent with data reported in other countries in the region following PCV introduction. In Peru, a recent study reported a 35% (95% CI; 8.6 − 53.8%) reduction in all-cause pneumonia mortality in children <1 y old in the period following PCV introduction, while in Chile, a nested case control study reported mortality reductions of 71.5% (95%CI: 9.0 − 91.8%) following PHiD-CV adoption in children <2 y of age.^18,19^ In Brazil, building upon a preexisting decline prior to PCV introduction, an estimated 12% reduction in all-cause pneumonia mortality was reported since PHiD-CV introduction in 2010, where notable differences were observed in different socioeconomic levels, with greatest benefit seen in municipalities with childhood poverty and lower maternal education.^20^

Changes in the incidence of all-cause pneumonia showed inconsistent patterns, with some evidence of declining incidence prior to any PCV introduction, and frequent and often large fluctuations in annual incidence estimates across the study period. In both Bogota and Medellin, reductions in the mean all-cause pneumonia incidence post vs. pre-PCV (66% and 40%) were observed, with a smaller reduction also seen in Cartagena (15%). Similar benefits in reducing all-cause pneumonia incidence have also been reported, with surveillance studies demonstrating reductions in hospitalization rates in Brazil,^21–24^ and in Chile.^19^ However, we found that in both Cali and Barranquilla a substantial increase in all-cause pneumonia incidence (of 125% and 78%, respectively) was observed in the post-PCV vs pre-PCV period comparisons, although in both cities incidence has declined since 2014. Looking at one of these cities in more detail, we found that in Cali, all-cause pneumonia incidence was relatively stable between 2008 − 2010, followed by an increase in both 2011 (the transition period) and 2012, stable between 2013 − 2014, after which the incidence declined. While we cannot readily account for this pattern, these data may reflect temporal variations in incidence of other contributing non-pneumococcal etiologies, while changes to reporting systems such as more complete surveillance in more recent years may also have had some effect; although we admit this is speculative.

For all-cause OM, we found in the post vs. pre-PCV period comparisons that the overall mean incidence was reduced Bogota and Medellin (by 51% and 42%, respectively). It must be noted that reductions in all-cause OM began prior to PCV UMV, year-to-year variation in all-cause OM was observed, and there are limited pre-PCV data available for some cities. While acknowledging the limitations of the current study, our observations are again somewhat consistent with reported data from the region. In a recent study in Brazilian infants <2 y, rate reductions in all-cause OM visits of 50.7% (95%CI: 42.2–59.2%) following PHiD-CV introduction have been reported,^25^ with reductions of 26.2% (95%CI: 16.9–34.4) in infants <1 y also reported in Peru,^18^ while a recent study from Chile has reported a 32% reduction in the frequency rate of hospital emergency department visits due to all-cause OM.^26^ In contrast, for Barranquilla, we found that the incidence of all-cause OM was substantially higher in the 2012–2016 post-PCV introduction period (by 69%), compared to the pre-PCV period (2008–2009), although when looking at specific later years the observed incidence has declined since 2014. For Cartagena and Cali, observed outliers in 2008 and possible underreporting in the following years did not permit us to calculate accurate percent reduction estimates.

Clearly, our results show divergent and often contradictory patterns of pneumonia and OM epidemiology, in particular when evaluating the period following switching to PHiD-CV introduction compared to the period prior to any PCV vaccine use (Figure 5). In this we see a broad picture of 1) Substantial reductions in all-cause pneumonia mortality and incidence, and in the incidence of OM in both Bogota and Medellin; 2) In both Barranquilla and Cali, while pneumonia mortality fell, pneumonia incidence increased substantially, and in Barranquilla, OM incidence also increased; 3) In Cartagena, there was no change in all-cause pneumonia mortality rates, while the incidence of pneumonia and OM was lower in the post-PHiD-CV introduction period.

These inconsistent patterns, and in the context of pneumonia, often contradictory results require further explanation, especially as vaccine uptake is relatively high (and in the period following PHID-CV introduction, particularly high in Barranquilla, where pneumonia incidence increased substantially). Evidently, our results for Bogota and Medellin are encouraging, highlighting consistent patterns of disease mortality and incidence reduction and clear benefits of the PHiD-CV immunization program in both cities. However, to some extent, there is no plausible explanation for the divergent disease patterns we see in Barranquilla and Cali, where we may have anticipated pneumonia incidence to fall, in line with the mortality reductions seen in both cities. This is particularly so for Barranquilla, where vaccine uptake was high since PHiD-CV introduction (Figures 4 and 5). While these may represent truly different patterns of disease outcomes, with greater infant pneumonia survival against a background of genuinely increasing incidence in these two cities, we feel it is more likely that our results reflect limitations inherent to our study, where our incidence data are dependent on the quality of the data reporting in the RIPS database. While the mortality data we obtained from DANE can be considered robust as in part it represents mortality surveillance across the overall infant population, the data on pneumonia and OM incidence were derived from RIPS, a claims-reimbursement database, which could be considered less reliable with potential for substantial bias. As described earlier when describing our data sources, the quality of reporting in RIPS from a surveillance perspective is subject to considerable uncertainty; under-reporting is well recognized, with variation in different areas of the country.^13,27,28^ What is less well understood is what impact changes on the quality of reporting may have had on the validity of longitudinal incidence estimations and comparisons. While a consistent pattern of under-reporting would not in itself impact on incidence rates and period reduction changes, improvements in the quality of reporting in more recent years (in terms of improved surveillance and also a broader coverage of the population) has potential to add substantial bias to incidence rate comparisons in different years and period rate comparisons.

The low incidence of all-cause pneumonia and OM observed in many cities across much of the study period may reflect such under-reporting. For example, the OM incidence estimates in Barranquilla, Cartagena, and Cali are substantially lower than those reported in Bogota and Medellin, and in general lower than may have been anticipated based on reported regional data.^4,29^ In addition, for much of the study period there was a higher number of pneumonia cases reported in RIPS than OM cases, with correspondingly higher incidence; a feature particular evident for Barranquilla, Cartagena, and Cali. One possible explanation for this may be due to differences in data reporting/data quality for infant pneumonia and OM. In Colombia, nearly all infants are hospitalized for pneumonia, with radiological diagnosis and it is possible to anticipate, accurate ICD-coding; in contrast for OM, the great majority are managed in primary care as an outpatient, and diagnosis and disease coding may be less robust, and cases may be under-reported. Furthermore, in Cartagena and Cali, the observed incidence of OM in 2008 (the first year of the study period) were markedly higher than that reported in later years with potential to distort period comparisons; hence, our approach not to include these cities in formal period comparisons for OM reductions; to some extent, this may also represent a signal of variation in disease reporting. These aspects of pneumonia and OM reporting in RIPS were unanticipated prior to our study, and perhaps highlight a need for improved surveillance and reporting for the incidence of all-cause pneumonia and OM in Colombia, as others have discussed.

Other factors may have also influenced our findings. Population demographic changes are not accounted for in official population statistics (such as an influx of non-registered residents from other municipalities into these cities) which may also impact on the precision of incidence/mortality estimates. As an ecological study, it was not possible to determine whether all-cause pneumonia or OM cases occurred in vaccinated children or in those either unvaccinated or with incomplete vaccination, and the study was not designed to assess any such direct effect of vaccination. In addition, we evaluated all-cause pneumonia and all-cause OM, and other infectious non-pneumococcal etiologies will have contributed to the pneumonia and OM burden we report. A prospective study, continuously monitoring pneumococcal disease-specific burden and incidence and mortality may help to assess the vaccine impact in future studies, coupled with improved disease surveillance systems. Finally, while the introduction of PCVs would have contributed to declining mortality and incidence trends observed in some cities, clearly other factors, e.g., better infant nutrition and parental education allied to improved public health-care services will also have had a substantial impact.

**Figure 6.**
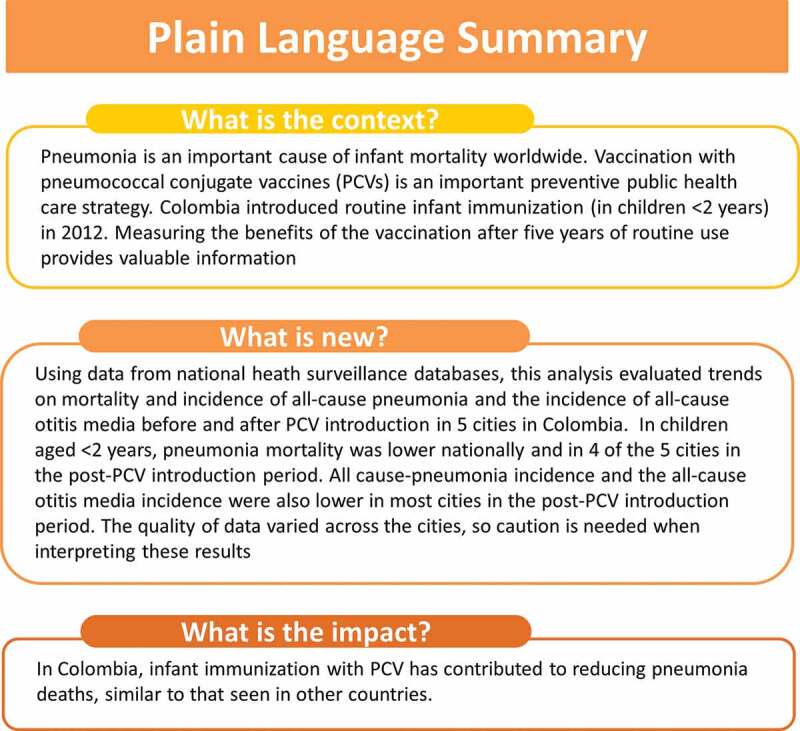
Focus on the patient

In conclusion, our data suggest there have been reductions in all-cause pneumonia mortality in the post-PCV period in Colombia; nationwide and in four of the largest cities. Reductions in all-cause pneumonia incidence estimates were observed in three cities, and the incidence of all-cause OM declined in two cities. Reductions in all three outcomes were observed in Bogota and Medellin, whereas the observed patterns of disease estimates were more variable and heterogeneous in Barranquilla, Cartagena, and Cali. These results need to be interpreted with caution, due to data limitations mentioned above. Figure 6 presents a plain-language summary of the context, outcomes and potential impact of this study for health-care providers.

•No legend.

## Supplementary Material

Supplemental Material
