# Supplementary material for: Trends in all-cause pneumonia and otitis media in children aged <2 years following pneumococcal conjugate vaccine introduction in Colombia
Source: Hum Vaccin Immunother. 2020 Sep 23;17(4):1173–80. doi: 10.1080/21645515.2020.1805990 (PMC8018459; doi:10.1080/21645515.2020.1805990)
Supplement: Supplemental Material [file KHVI_A_1805990_SM0158.docx]

**Supplementary Appendix**

**Trends in pneumonia and otitis media in children aged < 2 years following pneumococcal conjugate vaccine introduction in Colombia**

**Authors:**

Gabriel Carrasquilla^a^, Alexandra Porras-Ramírez^a,b^, Sandra Martinez^a^, Rodrigo DeAntonio^c^, Raghavendra Devadiga^d^, Carla Talarico^e^, Diana C. Caceres^f^, Maria M. Castrejon^g^, Patricia Juliao^g^

**Affiliations:**

^a^ ASIESALUD, Bogota, Colombia

^b^ Grupo de Medicina comunitaria y Salud colectiva, Universidad El Bosque, Bogotá, Colombia

^c^ Centro de Vacunación Internacional, S A CEVAXIN, Panama City, Panama

^d^ GSK, Biometrics, Bangalore, India

^e^ GSK, Rockville, USA

^f^ GSK, Bogotá, Colombia

^g^ GSK, Panama City, Panama

Address for correspondence:

Patricia Juliao. GSK, Epidemiology and Health Outcomes Latin America & the Caribbean, Panama City, Panama.

Email: [patricia.d.juliao@gsk.com](mailto:patricia.d.juliao@gsk.com).

Telephone: + 507 6619 7393

This appendix has been provided by the authors to give readers additional information about their work.

[**Supplementary Table 1. Study target population estimates for children <2 years in 5 selected cities and nationwide (2005-2016)** 3](#_Toc44176034)

[**Supplementary Table 2. Number of all-cause pneumonia deaths in children <2 years in five selected cities and nationwide (2005-2016)** 4](#_Toc44176035)

[**Supplementary Table 3. Number of deaths due to any cause in children <2 years in five selected cities and nationwide (2005-2016)** 5](#_Toc44176036)

[**Supplementary Table 4. Number of all-cause pneumonia cases in children <2 years in five selected cities (2008-2016)** 6](#_Toc44176037)

[**Supplementary Table 5. Number of otitis media cases in children <2 years in five selected cities (2008-2016)** 7](#_Toc44176038)

[**Supplementary Table 6. Overall trends by disease outcome in selected cities and nationwide for study period** 8](#_Toc44176039)

[**Supplemental Figure 1. Percent reductions in all-cause pneumonia and otitis media** 9](#_Toc44176040)

**Supplementary Table 1. Study target population estimates** **for children <2 years in 5 selected cities and nationwide (2005-2016)**

| Year | **Bogota** | **Barranquilla** | **Cartagena** | **Cali** | **Medellin** | **Nationwide** |
| --- | --- | --- | --- | --- | --- | --- |
| 2005 | 234,171 | 43,000 | 34,857 | 69,483 | 57,953 | 1,718,952 |
| 2006 | 233,731 | 42,627 | 34,656 | 69,563 | 57,678 | 1,708,561 |
| 2007 | 234,139 | 42,168 | 34,477 | 69,712 | 57,601 | 1,704,621 |
| 2008 | 235,244 | 41,691 | 34,364 | 69,904 | 57,736 | 1,704,843 |
| 2009 | 236,872 | 41,176 | 34,267 | 70,312 | 58,186 | 1,708,822 |
| 2010 | 238,667 | 40,703 | 34,140 | 70,819 | 58,909 | 1,714,922 |
| 2011 | 240,247 | 40,131 | 33,852 | 71,123 | 58,856 | 1,720,791 |
| 2012 | 241,434 | 39,629 | 33,491 | 71,236 | 58,790 | 1,725,890 |
| 2013 | 242,103 | 39,178 | 33,090 | 71,117 | 58,645 | 1,730,439 |
| 2014 | 242,338 | 38,816 | 32,733 | 70,929 | 58,378 | 1,735,511 |
| 2015 | 242,436 | 38,495 | 32,463 | 70,762 | 58,044 | 1,741,632 |
| 2016 | 243,156 | 38,177 | 32,312 | 70,807 | 58,022 | 1,747,273 |
| 2005-2016 | 2,864,538 | 485,791 | 404,702 | 845,767 | 698,798 | 20,662,257 |
| 2008-2016 | 2,162,497 | 357,996 | 300,712 | 637,009 | 525,566 | 15,530,123 |

**Supplementary Table 2. Number of all-cause pneumonia deaths in children <2 years in five selected cities and nationwide (2005-2016)**

| Year | **Bogota** | **Barranquilla** | **Cartagena** | **Cali** | **Medellin** | **Nationwide** |
| --- | --- | --- | --- | --- | --- | --- |
| 2005 | 103 | 38 | 34 | 43 | 40 | 750 |
| 2006 | 74 | 41 | 22 | 46 | 48 | 653 |
| 2007 | 89 | 40 | 20 | 44 | 52 | 705 |
| 2008 | 51 | 34 | 23 | 35 | 31 | 465 |
| 2009 | 41 | 19 | 15 | 26 | 45 | 437 |
| 2010 | 36 | 16 | 12 | 26 | 19 | 373 |
| 2011 | 33 | 26 | 20 | 32 | 39 | 360 |
| 2012 | 19 | 14 | 18 | 32 | 23 | 368 |
| 2013 | 15 | 19 | 29 | 23 | 17 | 328 |
| 2014 | 19 | 8 | 22 | 16 | 19 | 312 |
| 2015 | 19 | 24 | 24 | 14 | 17 | 328 |
| 2016 | 22 | 20 | 15 | 12 | 10 | 337 |
| 2005-2016 | 521 | 299 | 254 | 349 | 360 | 5,416 |

**Supplementary Table 3. Number of deaths due to any cause** **in children <2 years in five selected cities and nationwide (2005-2016)**

| Year | **Bogota** | **Barranquilla** | **Cartagena** | **Cali** | **Medellin** | **Nationwide** |
| --- | --- | --- | --- | --- | --- | --- |
| 2005 | 1,945 | 864 | 459 | 752 | 790 | 12,444 |
| 2006 | 1,811 | 896 | 325 | 754 | 793 | 12,266 |
| 2007 | 1,857 | 808 | 299 | 723 | 763 | 10,937 |
| 2008 | 1,810 | 676 | 404 | 784 | 763 | 11,396 |
| 2009 | 1,616 | 680 | 382 | 678 | 662 | 10,406 |
| 2010 | 1,480 | 537 | 280 | 636 | 584 | 9,095 |
| 2011 | 1,427 | 648 | 389 | 754 | 855 | 8,897 |
| 2012 | 1,441 | 533 | 404 | 565 | 555 | 8,990 |
| 2013 | 1,036 | 654 | 336 | 523 | 520 | 8,174 |
| 2014 | 1,226 | 683 | 306 | 498 | 477 | 7,919 |
| 2015 | 1,098 | 658 | 315 | 488 | 491 | 7,906 |
| 2016 | 1,029 | 660 | 315 | 468 | 419 | 7,296 |
| 2005-2016 | 17,766 | 8,297 | 4,214 | 7,623 | 7,672 | 115,726 |

**Supplementary Table 4. Number of all-cause pneumonia cases in children <2 years in five selected cities (2008-2016)**

| Year | **Bogota** | **Barranquilla** | **Cartagena** | **Cali** | **Medellin** |
| --- | --- | --- | --- | --- | --- |
| 2008 | 24,763 | 776 | 1,717 | 2,146 | 6,879 |
| 2009 | 14,393 | 623 | 1,305 | 1,929 | 4,715 |
| 2010 | 8,898 | 936 | 1,228 | 1,863 | 4,691 |
| 2011 | 10,305 | 806 | 1,524 | 3,731 | 3,678 |
| 2012 | 10,615 | 1,067 | 1,362 | 5,305 | 3,564 |
| 2013 | 8,638 | 1,419 | 1,219 | 5,273 | 2,425 |
| 2014 | 8,786 | 1,677 | 1,297 | 5,199 | 4,191 |
| 2015 | 8,386 | 910 | 1,585 | 3,973 | 3,273 |
| 2016 | 6,877 | 782 | 682 | 2,766 | 2,704 |
| 2008-2016 | 101,661 | 8,996 | 11,919 | 32,185 | 36,120 |

**Supplementary Table 5. Number of otitis media cases in children <2 years in five selected cities (2008-2016)**

| Year | **Bogota** | **Barranquilla** | **Cartagena** | **Cali** | **Medellin** |
| --- | --- | --- | --- | --- | --- |
| 2008 | 23,096 | 173 | 3,421 | 7,733 | 7,314 |
| 2009 | 22,304 | 690 | 562 | 1,658 | 8,449 |
| 2010 | 16,610 | 771 | 667 | 1,189 | 5,319 |
| 2011 | 17,934 | 534 | 730 | 2,213 | 5,140 |
| 2012 | 15,278 | 733 | 635 | 2,369 | 3,247 |
| 2013 | 11,559 | 685 | 555 | 2,183 | 3,619 |
| 2014 | 10,977 | 1096 | 741 | 2,518 | 5,127 |
| 2015 | 12,433 | 881 | 605 | 2,006 | 4,793 |
| 2016 | 7,916 | 567 | 361 | 1,234 | 3,587 |
| 2008-2016 | 138,107 | 6,130 | 3,421 | 23,103 | 46,595 |

**Supplementary Table 6. Overall trends by disease outcome in selected cities and nationwide for study period**

| **Mortality estimates (2005-2016 period)** | | | | |
| --- | --- | --- | --- | --- |
|  | **All-cause pneumonia mortality** | | **Overall (all-cause) mortality** | |
|  | **Yearly % Reduction (95%CI)*** | **p-value*** | **Yearly % Reduction (95%CI)*** | **p-value*** |
| National | 8.9 (6.3, 11.4) | <0.0001 | 5.0 (4.5, 5.6) | <0.0001 |
| Bogota | 16.8 (13.8, 19.7) | <.00001 | 6.3 (5.3, 7.3) | <0.0001 |
| Barranquilla | 7.5 (3.1, 11.7) | 0.001 | 1.5 (-0.3, 3.4) | 0.1068 |
| Cartagena | 1.4 (-3.0, 5.5) | 0.5349 | 1.1 (-1.1, 3.2) | 0.3362 |
| Cali | 10.3 (7.4,15.3) | <0.0001 | 5.0 (3.7, 6.2) | <0.0001 |
| Medellin | 11.4 (7.4, 15.3) | <0.0001 | 5.7 (3.8, 7.5) | <0.0001 |
| **Incidence estimates (2008-2016 period)** | | | | |
|  | **All-cause pneumonia** | | **Otitis media** | |
|  | **Yearly % Reduction (95%CI)*** | **p-value*** | **Yearly % Reduction (95%CI)*** | **p-value*** |
| Bogota | 11.9 (7.6, 16.1) | <.0001 | 11.7 (9.3, 14.0) | <0.0001 |
| Barranquilla | -7.2 (-15.3, 0.4) | 0.0636 | -10.4 (-22.6, 0.6) | 0.065 |
| Cartagena | 3.6 (-1.6, 8.6) | 0.1741 | 16.2 (7.1, 24.5)† | 0.0008 |
| Cali | -10.5 (-21.2, -0.8) | 0.0331 | 12 (2.0, 20.9) † | 0.0197 |
| Medellin | 8.7 (4.5, 12.6) | <0.0001 | 8.0 (3.1, 12.6) | 0.0017 |
| *Overall trends (Yearly %reduction, 95%CI and associated p-values) were calculated using negative binomial regression; calculated by log (No. of cases [or death]) = log (population projections) + intercept + β1Year  † Outlier detected in 2008 that may bias trend results | | | | |

**Supplemental Figure 1. Percent reductions in all-cause pneumonia and otitis media**


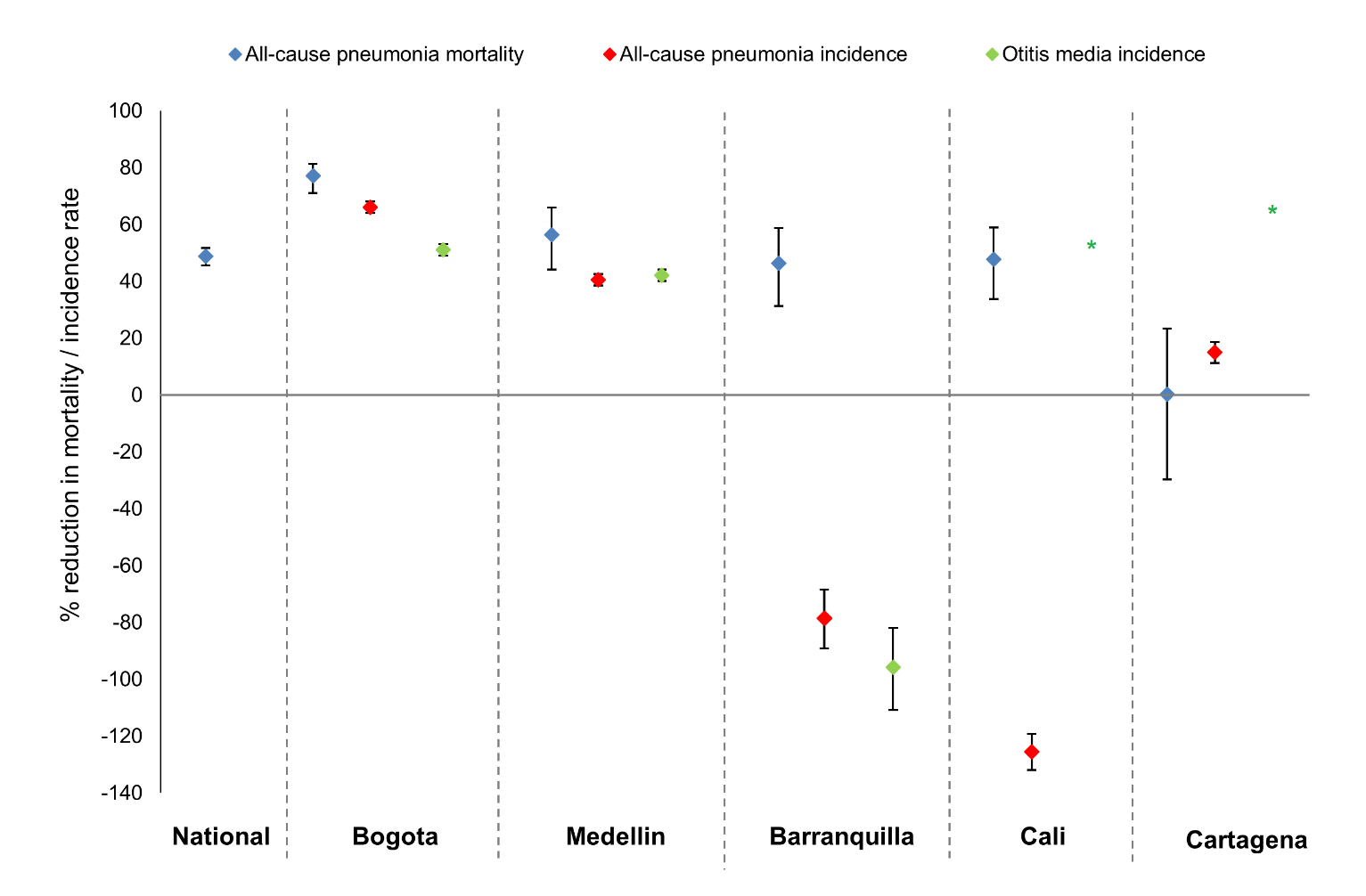


Data represents percent reductions in mortality/incidence rates in the period following PHiD-CV introduction compared with those prior to use of any PCV.

* Due to substantial biases observed with outliers and possible underreporting of otitis media, these cities were not included in the formal analyses for otitis media incidence reductions.
